# Fabrication of a Chitosan–Gelatin/Polylactic Acid Bilayer Active Film Loaded with Tannic Acid for Enhancing Shelf-Life of Refrigerated Baby Clams

**DOI:** 10.3390/foods14223934

**Published:** 2025-11-17

**Authors:** Arunachalasivamani Ponnusamy, Suriya Palamae, Thummanoon Prodpran, Jun Tae Kim, Bin Zhang, Lukai Ma, Soottawat Benjakul

**Affiliations:** 1 International Center of Excellence in Seafood Science and Innovation, Faculty of Agro-Industry, Prince of Songkla University, Hat Yai 90110, Songkhla, Thailand; 6511030004@email.psu.ac.th (A.P.); suriya.pal@psu.ac.th (S.P.); thummanoon.p@psu.ac.th (T.P.); 2 Center of Excellence in Bio-Based Materials and Packaging Innovation, Faculty of Agro-Industry, Prince of Songkla University, Hat Yai 90110, Songkhla, Thailand; 3 BioNanocomposite Research Center, Department of Food and Nutrition, Kyung Hee University, 26 Kyungheedae-ro, Dongdaemun-gu, Seoul 02447, Republic of Korea; jtkim92@khu.ac.kr; 4 Key Laboratory of Health Risk Factors for Seafood of Zhejiang Province, College of Food Science and Pharmacy, Zhejiang Ocean University, Zhoushan 316022, China; zhangbin@zjou.edn.cn; 5 Key Laboratory of Green Processing and Intelligent Manufacturing of Lingnan Specialty Food of Ministry and Rural Affairs, College of Light Industry and Food, Zhongkai University of Agriculture and Engineering, Guangzhou 510225, China; malukai@zhku.edu.cn

**Keywords:** storage stability, bioactive compound, release kinetics, bivalves, microbiology

## Abstract

Active bilayer (BL) packaging films were developed by depositing chitosan/fish gelatin blend containing tannic acid (TA) at varying levels (1, 3, and 5%; *w*/*w*) onto a polylactic acid layer. Augmenting TA levels enhanced strength (17.27 MPa to 27.57 MPa) but reduced flexibility (85.03% to ~38%) due to enhanced polymer cross-linking induced by TA, as confirmed by FTIR. The films exhibited exceptional UV-blocking capabilities, in which UVB protection reached 98.34% and 100% for films with 1% and 3% TA, respectively. SEM micrographs revealed uniform dispersion of TA with defect-free matrices. The antioxidant activity of the films upsurged with rising TA levels. When baby clam edible portions (BC-EP) were packed in pouches made with BL films, the pouches containing 5% TA most effectively slowed lipid oxidation and inhibited spoilage during 12 days of refrigerated storage. Total viable count, psychrotrophic bacteria count, and counts of specific spoilage organisms were decreased. Reductions in spoilage bacteria including *Shewanella* and *Pseudomonas* and dominance of lactic acid bacteria were confirmed using next-generation sequencing analysis. The release kinetics of TA followed Fickian diffusion, enabling sustained antioxidant and antimicrobial action. TA-containing pouches therefore effectively extended the shelf life of BC-EP through synergistic barrier, antioxidant, and antimicrobial mechanisms, offering a sustainable alternative to conventional plastic packaging.

## 1. Introduction

Seafood is an essential source of high-quality protein and other nutrients; however, its high perishability often results in significant postharvest losses. Among various seafood products, baby clams, or undulated surf clams (*Paphia undulata*), are a commercially valuable bivalve that inhabits shallow marine environments as a benthic species. They are widely distributed along Thailand’s Southern coast, Southeast Asia, and Southern China, and they have recently gained growing economic importance. Baby clams are valued for their unique flavor, high protein content, low fat, and certain medicinal properties, making them highly desirable for consumers [1,2]. However, due to their high water activity, abundant glycogen, and near-neutral pH, baby clams undergo spoilage. Their shelf life is only a few days under refrigeration. Their filter-feeding nature also predisposes them to microbial contamination, which further accelerates spoilage. This rapid deterioration poses major challenges for fishermen, processors, and the seafood industry.

Spoilage of clams is closely associated with the proliferation of specific microorganisms, including *Vibrio* spp., *Shewanella* spp., *Aeromonas* spp., and *Pseudomonas* spp. [3]. Their metabolic activity accelerates the breakdown of proteins, releasing volatile nitrogenous compounds such as ammonia, trimethylamine, and dimethylamine, which contribute to off-odors and are generally measured as total volatile basic nitrogen (TVBN) [4]. Oxidative processes are another phenomenon resulting in the formation of primary and secondary lipid oxidation products, typically assessed through peroxide value (PV) and thiobarbituric acid reactive substances (TBARSs). Collectively, these changes lead to off-odor, textural deterioration, reduced sensorial acceptability and compromised food safety [5].

To lengthen the shelf life of clams and other seafood products, active packaging has attracted increasing attention. Packaging functions as the primary barrier against physical damage, chemical deterioration, and microbial contamination. Innovations in packaging technology are therefore critical for enhancing seafood preservation efficacy. Traditional films such as polyethylene (PE) provide only passive protection and do not actively inhibit microbial or oxidative deterioration. In contrast, biopolymer-based films have become a sustainable, biodegradable alternative with desirable functional properties and bioactivites. Chitosan (CS), a natural polysaccharide, is well recognized for its antimicrobial activity and film-forming ability, while fish gelatin (FG) contributes to elasticity and transparency [6]. When blended, CS and FG form cohesive films with balanced mechanical and barrier properties. However, single-layer films often have limited strength, low hydrophobicity, and poor barrier capacity compared to petroleum-based plastics. Multilayer film systems, such as bilayers, have emerged as an innovative solution by combining distinct functional layers to overcome these drawbacks. For instance, the use of a hydrophobic outer layer like polylactic acid (PLA) can significantly improve moisture resistance, while a bioactive inner layer can provide direct protection to the food [7]. Consequently, structural integrity, water resistance, and the stability of incorporated active compounds can be enhanced [8].

Incorporation of bioactive molecules into the active layer of such bilayer films further expands their functionality in controlling spoilage microorganisms and slowing lipid oxidation of packed foods [7]. Tannic acid (TA), a naturally occurring plant polyphenol, is particularly attractive due to its strong antioxidant and antimicrobial activities, UV-shielding capacity, and ability to interact with proteins and polysaccharides through hydrogen bonding and hydrophobic interactions [9]. These interactions improve compactness, mechanical stability, and reduce permeability to gases and water vapor of resulting films. Moreover, TA can mitigate oxidative and microbial spoilage by scavenging free radicals and inhibiting bacterial enzymes [10]. Therefore, films that can delay microbial proliferation and suppress oxidative deterioration are essential for prolonging the quality of clams under refrigerated storage. Despite these advantages, research on the incorporation of TA into bilayer films designed specifically for seafood preservation remains limited. Most previous studies focused on single-layer films or other bioactive compounds [11], leaving a gap in understanding the synergistic benefits of a TA-loaded bioactive layer combined with a protective PLA layer with the excellent barrier.

In this context, the present study aimed to develop bilayer films comprising a CS-GE blend film incorporated with TA at varying concentrations (0–5% *w*/*w*) as the inner layer, whereas PLA film served as the outer layer. The films were characterized based on physicochemical, mechanical, optical, thermal, and morphological properties. Their application in the form of a pouch to preserve refrigerated baby clams was also investigated, in which microbial community features via next-generation sequencing, microbial counts, and chemical quality indices were monitored throughout 12 days of storage. This work highlights the novelty of utilizing a TA-fortified bilayer system for seafood preservation, a previously understudied topic of research.

## 2. Materials and Methods

### 2.1. Chemicals and Raw Material

Polylactic acid pellets (PLA) were procured from Nature Work Co., Ltd. (Blair, NE, USA). Chitosan (CS) (molecular weight ~210 kDa; degree of deacetylation of ~82%; viscosity of 1000–2000 cps) was acquired from Sigma-Aldrich (St. Louis, MO, USA). Fish gelatin powder (FG) (molecular weight ~51 × kDa; moisture content of 9.99%) was obtained from Vinh Hoan Corp. (Cao Lanh, Dong Thap, Vietnam). Acetic acid was purchased from RCI lab scan Limited (Bangkok, Thailand). Tannic acid (TA) and chemicals for antioxidant assays (analytical grade) were acquired from Loba Chemie (Mumbai, India), Sigma-Aldrich (St. Louis, MO, USA), while the media and chemicals used for antimicrobial assays were procured from Oxoid (Thermo Fischer Scientific, Waltham, MA, USA).

### 2.2. Preparation of PLA/CSFG Bilayer Films Loaded with TA at Various Concentrations

Bilayer films were prepared using a solvent-casting method, detailed by Ponnusamy, Rajasekaran, Tagrida, Prodpran, Kim, and Benjakul [8] with slight modification. For the base layer preparation, PLA pellets were firstly dispersed in chloroform to obtain a concentration of 3.25% (*w*/*v*), followed by continuous magnetic stirring at ambient temperature (28 ± 2 °C) until a homogeneous solution was obtained. The PLA solution was then evenly cast onto aluminum trays (20 cm × 20 cm) and allowed to dry slowly at ambient temperature (28 ± 2 °C) for 12 h to form a solid, solvent-free base layer before the second layer was cast. For preparation of the second layer, chitosan (CS, 2% *w*/*v*) and fish gelatin (FG, 2% *w*/*v*) were separately dissolved in 1% (*v*/*v*) acetic acid and distilled water at 45 °C, respectively. Once completely dissolved, the CS and FG solutions were mixed at a 1:1 ratio, a proportion previously shown to yield films with balanced mechanical and barrier properties [12]. Glycerol (25% *w*/*w*, based on the total CS and FG polymer mass) was added as a plasticizer to improve flexibility and reduce film shrinkage. TA was incorporated at varying concentrations (0, 1, 3, and 5% (*w*/*w*) based on polymer blend. Thereafter, film forming solution (FF-S) was then cast over the pre-formed PLA layer to create the bilayer structure. The resulting films were designated as BL (control), BL^1%TA^, BL^3%TA^, and BL^5%TA^. After drying for 24 h in air at ambient temperature, the films were carefully peeled from the trays and conditioned for 48 h in an environmental chamber (WTB Binder, Tutt Lingen, Germany) at 25 °C and 50% relative humidity (RH) prior to characterization.

### 2.3. Characterization of Active Bilayer Films

#### 2.3.1. Thickness, Color, and Light Barrier Properties

The thickness of the films was determined at seven randomly selected locations using a digital micrometer (Mitutoyo Corp., Model IDC112PM, Kawasaki-shi, Japan) with a precision of ±1 µm. The mean value was calculated, representing the thickness of each film. The color of the prepared films was quantitatively analyzed using a colorimeter (Hunterlab, Reston, Virginia, USA) based on the CIE color system. L*: lightness (values between 0 and 100), a*: redness (+)/greenness (−), b*: yellowness (+)/blueness (−), and total color difference (Δ*E*) of the samples were calculated against a standard white plate (L* = 92.83, a* = −1.23, and b* = 0.48).

The light barrier capacity of the films was assessed using a UV–Vis spectrophotometer (UV-1800, Shimadzu, Kyoto, Japan). Film samples were scanned over a wavelength range of 200–800 nm, with air serving as the reference. To determine film opacity, the absorbance at 600 nm (A_600_) was measured, and the opacity was calculated using the following formula:

$$\mathrm{Opacity}\;\mathrm{value}\;=\;\frac{A_{600}}{X}$$

where A_600_ represents the absorbance at 600 nm and X is film thickness in mm. Higher opacity values were associated with the increased light-blocking ability.

#### 2.3.2. Mechanical Characteristics and Water Vapor Permeability (WVP)

The tensile strength (TS) and elongation at break (EAB) of the films were measured using a universal testing machine (Lloyd Instrument, Hampshire, UK). Rectangular film specimens (50 × 20 mm^2^) were clamped with an initial grip separation of 40 mm and subjected to uniaxial tension at a constant crosshead speed of 30 mm/min until fracture. The TS (MPa) was calculated as the maximum load at rupture divided by the original cross-sectional area of the specimen, while EAB (%) was determined as the percentage increase in length at the point of rupture relative to the initial gauge length.

The water vapor permeability (WVP) of the films was determined using a gravimetric method. Circular film samples (3 cm in diameter) were mounted onto aluminum permeation cups containing anhydrous silica gel (0% RH) in which the PLA layer of the BL was facing the external environment. The edges were sealed with rubber gaskets to prevent vapor leakage and ensure an airtight system. The cups were then placed in an environmental chamber (WTB Binder, Tutt Lingen, Germany) maintained at 25 ± 1 °C and 50 ± 2% RH. The mass of each cup was recorded at 1 h intervals over a 10 h period using an analytical balance with a precision of ±0.0001 g. Water vapor permeability (WVP, g·m^−1^·s^−1^·Pa^−1^) was subsequently computed using the standard equation described below:

$$\mathrm{WVP}\;({\mathrm{gm}}^{-1}s^{-1}{\mathrm{Pa}}^{-1})\;=\;\frac{w\times \;l}{t\;\times \;A\;\times \;∆P}$$

where w is the weight gain of the permeation cup (g); l represents the average thickness of the film (m); t is the elapsed time (s); A is the exposed area of the film (m^2^), and Δ*P* is the vapor pressure difference across the film samples (Pa).

#### 2.3.3. Attenuated Total Reflection–Fourier Transform Infrared (ATR-FTIR) Spectroscopic Spectra

The functional groups and molecular interactions between polymers and TA within the films were characterized using Fourier transform infrared spectroscopy (FTIR; Model Equinox 55, Bruker Co., Ettlingen, Germany) equipped with an attenuated total reflectance (ATR) accessory with a platinum crystal, following the method described by Ponnusamy, Rajasekaran, Tagrida, Prodpran, Kim, and Benjakul [8]. Prior to analysis, film samples were carefully placed on the ATR crystal to ensure consistent contact. Spectra were acquired at 25 ± 0.5 °C across the mid-infrared range (4000–400 cm^−1^), with 32 scans collected at a resolution of 4 cm^−1^ to enhance the signal-to-noise ratio. All spectra were normalized to account for possible variations in sample thickness and contact pressure. The data were then analyzed to identify characteristic functional groups and to assess the molecular interactions within the film matrix.

#### 2.3.4. Antioxidant Activities

The antioxidant potential of the films was assessed using in vitro assays [8]. Film samples (100 ± 1 mg) were accurately weighed and suspended in 10 mL of distilled water under magnetic stirring for 16 h at 25 ± 1 °C. The suspensions were then centrifuged at 2800× *g* for 30 min at room temperature to obtain clear supernatants for further analysis. Although aqueous extraction may not fully solubilize all phenolic compounds compared to organic solvents (ethanol and methanol), it was selected to simulate the release of active compounds into a high-moisture food system like seafood. The radical scavenging activities (RSA) against 2,2-diphenyl-1-picrylhydrazyl (DPPH) and 2,2′-azino-bis(3-ethylbenzothiazoline-6-sulfonic acid) (ABTS) radicals, as well as the ferric-reducing antioxidant power (FRAP), were determined and expressed as millimoles of Trolox equivalents per gram of film (mmol TE/g). Additionally, the metal chelating capacity was determined and reported as millimoles of EDTA equivalents per gram of film (mmol EE/g).

#### 2.3.5. Scanning Electron Microscope (SEM) Images

The surface and cross-sectional microstructures of the films were analyzed using a field emission scanning electron microscope (SU3900, Hitachi, Tokyo, Japan). Film samples were mounted on bronze stubs with conductive carbon tape and sputter-coated with a thin gold layer (~10 nm) using an SPI Module Sputter Coater to enhance electron conductivity. For cross-sectional imaging, the films were cryo-fractured in liquid nitrogen to obtain clean fracture surfaces while minimizing structural deformation before visualization.

Micrographs were acquired under high vacuum at an acceleration voltage of up to 20 kV. Images at multiple magnifications were captured to evaluate both surface morphology and internal layer structure.

#### 2.3.6. Thermogravimetric Property

The thermal properties of the film samples (~5–10 mg) were evaluated using a thermal analyzer (STA 8000, PerkinElmer, Norwalk, CT, USA). Samples were heated from 30 to 700 °C at a rate of 10 °C/min under a nitrogen atmosphere with a flow rate of 20 mL/min. Thermogravimetric analysis (TGA) was employed to determine the weight loss of the bilayer films, while derivative thermogravimetric analysis (DTG) was used to assess specific thermal degradation patterns. Films with and without TA incorporation were examined.

#### 2.3.7. Release Profile and Kinetics

Film samples (2 × 2 cm^2^) were immersed in 10 mL of 0.05 M phosphate buffer (PB; pH 6.0) and incubated at room temperature (25 ± 2 °C; RT) to assess the release kinetics of TA from the BL matrix. Aliquots were collected at specified time intervals over a total period of 50 h, following the procedure of Ponnusamy, Rajasekaran, Tagrida, Prodpran, Kim, and Benjakul [8].

The cumulative release of TA was determined by fitting the experimental data to various kinetic models, including zero-order, Higuchi, and Korsmeyer–Peppas models. Release profiles of films containing TA at concentrations of 1, 3, and 5% (*w*/*w*) were plotted using R software (Ver: RStudio 2025.09.1+401, Posit). Baseline corrections were carried out by subtracting the absorption values of control films at corresponding time points. Kinetic parameters, including the rate constant (k), release exponent (n), and correlation coefficients (R^2^), were calculated to elucidate the release mechanism of TA from the film matrix.

### 2.4. Preparation of Pouch for Packaging Baby Clams

Pouches made of BL film were fabricated using an impulse sealer (Model ME-300HIM, S⋅N.MARK Ltd., Pak Kret, Nonthaburi, Thailand). For pouch formation, two CS-FG blend films with or without TA were faced inward. The films were cut into dimensions of 6 × 7 cm^2^ and sealed on three sides at 150 ± 0.5 °C for 1.25 s per side, followed by a 3.5 s cooling period. All pouches had 2 mm-wide seals. Commercial polyethylene (PE) pouches with the same dimensions were prepared in an identical manner and used as the control.

Baby clams (*Paphia undalata*), averaging 6.6 ± 1.5 g, embedded in crushed ice were obtained from a local fish market in Hat Yai, Songkhla, Thailand. Clams were shucked manually and immediately placed in PE bags before embedding in an ice bath to maintain freshness. The edible portion of each clam (BC-EP) was portioned and individually placed into the prepared pouches. Residual air was manually removed before hermetically sealing each package. All packaged samples were stored under refrigerated conditions (4 ± 1 °C) for 12 days. Comprehensive quality assessments were performed at 3-day intervals. Packages were randomly selected at each sampling point for analysis.

### 2.5. Quality Assessment of BC-EP

The pH of BC-EP samples was determined using a calibrated pH meter (Sartorius, New York, NY, USA). Prior to measurement, samples were homogenized with distilled water at a 1:5 (*w*/*v*) ratio, following the procedure of Palamae, Saetang, Yingkajorn, Zhao, Zhang, Kim, and Benjakul [3]. Lipid oxidation was assessed through peroxide value (PV) and thiobarbituric acid reactive substance (TBARS) assays, as described by Ponnusamy, Niluswan, Prodpran, Kim, Rhim, and Benjakul [7]. PV was expressed as milligrams of cumene hydroperoxide equivalents per kilogram of BC-EP, while TBARS value was reported as milligrams of malondialdehyde equivalents per kilogram of BC-EP. The total volatile basic nitrogen (TVB-N) content was determined using the Conway micro-diffusion method and expressed as milligrams of nitrogen per 100 g of BC-EP.

Microbiological analysis was performed by homogenizing 5 g of BC-EP samples with 45 mL of sterile saline solution (0.85% NaCl) using a stomacher (M400, Seward, UK) at 220 rpm for 1 min. Serial decimal dilutions were prepared from the homogenate, and aliquots were spread-plated on selective media. Total viable counts (TVCs) were enumerated on plate count agar after incubation at 37 °C for 24 h. Psychrotrophic bacteria counts (PBCs) were assessed by incubating plates at 4 °C for 7 days. Specific bacterial groups were quantified using selective media: thiosulfate-citrate-bile salts-sucrose (TCBS) agar for *Vibrio* spp., tryptic soy iron (TSI) agar for *Shewanella* spp., and *Aeromonas* selective agar for *Aeromonas* spp. All microorganisms tested were incubated at 37 °C for 24 h. *Pseudomonas* counts were determined on *Pseudomonas* base agar with cetrimide-Fucidin-cephalosporin supplement and incubated at 25 °C for 24 h. All microbial counts were expressed as log colony-forming units/g BC-EP (log CFU/g).

### 2.6. Next-Generation Sequencing

Bacterial community profiling was conducted using 16S rRNA gene next-generation sequencing (NGS), as described previously by Palamae, Saetang, Yingkajorn, Zhao, Zhang, Kim, and Benjakul [3]. BC-EP samples collected on day 0 and after 12 days of refrigerated storage (packaged in PE bags and BL^5%TA^) were subjected to analysis. Genomic DNA was extracted using the ZymoBIOMICS^®^-96 MagBead DNA Kit (Zymo Research, Irvine, CA, USA) according to the manufacturer’s protocol. The hypervariable V3–V4 regions of the 16S rRNA gene were amplified via polymerase chain reaction (PCR). Amplicon sequencing was performed on the Illumina^®^ MiSeq™ platform using a v3 reagent kit (600 cycles), with a 10% PhiX spike-in included to improve sequencing accuracy. Taxonomic assignment was carried out using Uclust within the QIIME v.1.9.1 pipeline, while microbial community composition was visualized using the Zymo Research Database.

### 2.7. Data Analysis

The experimental design was conducted using a completely randomized design (CRD), which was implemented throughout all experiments. The collected data were analyzed using one-way analysis of variance (ANOVA) to determine significant differences among treatment groups. For post hoc analysis, Duncan’s Multiple Range Test was employed for comparisons of means when ANOVA indicated significant differences (*p* < 0.05). All statistical computations were performed using SPSS software (Version 25.0, IBM Corporation, Armonk, NY, USA). Results are presented as mean ± standard deviation (SD) from triplicate measurements. Significance was considered at *p* < 0.05.

## 3. Results and Discussion

### 3.1. Effects of Different Levels of TA on Properties of Bilayer Films

#### 3.1.1. Film Thickness, Mechanical, and Barrier Properties

Thickness is a crucial factor, as it affects the mechanical and barrier properties of the composite films. Table 1 provides the mechanical and barrier properties of BL films containing TA. The thickness of the BL films remained remarkably consistent across all formulations, ranging from ~100.0 to 103.5 µm. The addition of TA, even at 5% (*w*/*w*), did not substantially alter the film thickness (*p* > 0.05). This indicated that TA integrated into the polymer CS/FG matrix did not cause major changes in the film network or phase separation that could lead to inconsistent casting and drying [11]. This consistent thickness reflected that there was no confounding variable in the interpretation of mechanical and barrier properties. Previously, Sutharsan et al. [13] observed similar results for CS films upon the addition of catechin and quercetin at low to moderate concentrations.

TS, a measure of the film’s resistance to breaking under tension, showed a remarkable improvement from 17.27 MPa for the BL film to 27.57 MPa for the film with 5% TA (Table 1). This pronounced enhancement was primarily attributed to the potent cross-linking ability of TA, which served as a reinforcing compound between the film components. TA is a polyphenol rich in hydroxyl groups (-OH) that can form strong intermolecular interactions, specifically hydrogen bonds with the amino groups in CS as well as carboxyl (CO) and amino (NH_3_) groups in FG [11]. This proposed cross-linking was later confirmed by FTIR analysis, which showed band broadening and shifts of peak representing the formation of hydrogen bonds (Section 3.1.3). This cross-linking created a denser and more cohesive three-dimensional network within the CS-FG blend, thereby increasing the mechanical integrity and the force required to break the film. The notable difference in TS was observed with increasing TA concentrations, likely due to enhanced CS-FG and TA interactions (*p* < 0.05). Lee et al. [14] also reported that CS films incorporated with TA showed a similar trend, where TS upsurged with the addition of TA. Yu et al. [15] documented that TA acted as a cross-linker, significantly improving FG film’s strength. Moreover, the inclusion of TA in the CS-FG layer plausibly acted as a binder to attach to the PLA layer via H-bonding. Conversely, EAB, which indicates the flexibility and ability of the films to stretch, decreased dramatically with the addition of TA. The EAB dropped from 85.03% for the control to approximately 38% for films supplemented with 3% and 5% TA (Table 1). This inverse relationship between TS and EAB is a common characteristic of polymer cross-linking in the film matrix. The cross-linking enhanced strength and rigidity by restricting polymer chain mobility and simultaneously reduced the film’s extensibility [16]. This may have been due to the excessive crosslinking by TA. As a result, the polymer chains became tightly bound and could not stretch easily when stress was applied. This led to a more brittle film that was broken after less deformation occurred. The results were consistent with studies on FG films containing TA, which showed a significant reduction in elongation [15].

The WVP measures the ability of films to resist the transmission of water vapor. The WVP values of the bilayer films were notably lower than those reported for monolayer CS-FG films, which typically range from 1.53 × 10^−11^ g·m^−1^·s^−1^·Pa^−1^ [12], confirming that the hydrophobic PLA base layer provided a superior barrier to water vapor. The WVP showed a slight decreasing trend with augmenting TA concentration. This could be due to the cross-linked network, which created a more tortuous path for water vapor molecules to diffuse through. Furthermore, the hydrophobic nature of the aromatic rings in TA could slightly reduce the overall hydrophilicity of the CS-FG blend. However, TA improved the water vapor barrier only modestly within this concentration range (1–5% *w*/*w* of polymer) and in this specific bilayer structure, as indicated by non-significant differences in WVP (Table 1). Moreover, the dominant barrier property was likely provided by the hydrophobic PLA base layer, while the CS-FG containing TA served as the active packaging layer.

#### 3.1.2. Color, Appearance, and Optical Properties

The incorporation of TA significantly affected the color, optical, and UV-barrier properties of the BL films, as shown in Table 2. The BL film had a very high L* value (89.59), indicating a light and translucent film, which was consistent with the previous report [8]. The addition of TA caused a significant and progressive decrease in L* to 78.63 for BL^5%TA^ (*p* < 0.05). This indicated that the films became progressively darker with increasing TA concentration. This darkening resulted from the intrinsic brown coloration of TA, which imparted a yellowish-brown hue to the film. The a* value shifted dramatically from negative (−1.53) to positive (4.49), suggesting a slight greenish tint to a distinct reddish hue. Similarly, the b* value increased substantially from 3.17 to 10.80, indicating a strong increase in yellowness. These shifts were attributed to the incorporation of the intensely colored TA having reddish brown tones. The total color change (Δ*E*) increased dramatically from 4.25 to 18.48. A Δ*E* value above 2 is considered perceptible to the human eye, and a value above 12 is considered a very distinct and obvious color change [17]. The values observed in the present study (11.82 to 18.48) confirmed that the films with TA were visually distinct from the control and the color intensity was directly proportional to TA concentration.

CS films incorporated with TA showed a similar dose-dependent decrease in L* and increase in a* and b* values, resulting in high Δ*E* values, making the films darker and more yellow-brown [14]. Similarly, FG-based films containing tannin-rich grape seed extract exhibited reduced lightness and increased yellowness, corroborating the present findings. The increases in yellowness and redness were also obtained since polyphenolic compounds drastically alter the optical properties of biopolymer films [18]. Furthermore, the uniform coloration indicated the homogenous dispersion of TA throughout the film.

**Figure 1:**
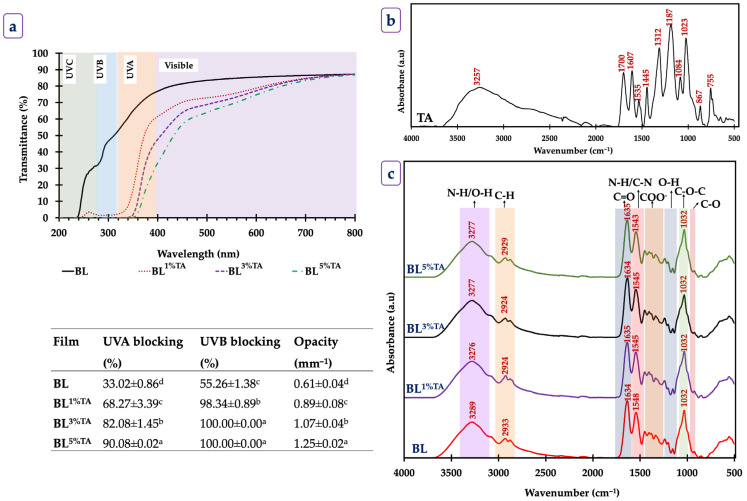
UV-visible transmittance spectra (**a**), FTIR spectrum of tannic acid (**b**), and spectra of bilayer films (**c**) based on chitosan/fish gelatin loaded without and with tannic acid at different levels deposited on polylactic acid film. Values are mean ± standard deviation (*n* = 3). Different superscripts within the same column indicate significant differences (*p* < 0.05). BL: bilayer films, TA superscript: tannic acid; the numbers in superscript denote the percentage of TA loaded in the CS/FG film.

BL films exhibited their ability to block UV radiation. The BL film offered minimal UVA protection (33.02%). It was noted that 1% TA incorporation showed approximately 2-fold higher efficiency in UV blocking (68.27%) than the control film. The protecting effect was TA concentration-dependent, in which BL^3%TA^ and BL^5%TA^ achieved excellent UVA blocking rates of 82.08% and 90.08%, respectively. The enhancement in UVB blocking was more prominent. The BL film blocked 55.26% of UVB light, and the addition of TA (1%) increased the blocking efficiency to 98.34%. The films supplemented with 3% and 5% TA achieved complete (100%) UVB blocking. This exceptional UV barrier property was due to the high polyphenolic content of TA. The strong UV absorption by TA is directly attributed to its aromatic rings and conjugated π-electron system, which efficiently absorb high-energy UV radiation [9]. These compounds, possessing aromatic rings with conjugated double bonds, acted as a powerful internal UV filter, absorbing high-energy UV radiation [19]. The formation of complex interactions between TA and the amino groups of CS and FG might also create a denser network that further impedes UV light transmission. Previously, Ponnusamy, Rajasekaran, Tagrida, Prodpran, Kim, and Benjakul [8] observed reduced UV light transmittance after incorporation of epigallocatechin gallate (EGCG) in BL films. CS-furcellaran films with nettle extract had a maximum UVB blocking of ~85% [20]. The result suggests that TA acted as a highly efficient natural UV absorber with potential to protect light-sensitive food products from oxidation. All the BL films with and without TA were slightly opaque, and light transmission was reduced in the visible region (Figure 1).

The opacity of the films significantly increased with the addition of TA, from 0.61 mm^−1^ for the BL to 1.25 mm^−1^ for BL^5%TA^ (*p* < 0.05). This increase in opacity was directly linked to the dark color of TA, which was able to absorb visible light. In addition, the light-scattering effect caused by the dispersion of TA particles within the polymer matrix, and potential micro-aggregation also caused an increase in the opacity of the films. Overall, increasing TA concentration enhanced the films’ visual opacity and UV-barrier capacity through combined pigmentary and structural effects, thereby improving their potential to protect light-sensitive foods.

#### 3.1.3. FTIR Spectra and Microstructure

The FTIR spectrum of pure TA is shown in Figure 1b. For the TA spectrum, the broad absorption band at ~3257 cm^−1^ corresponds to O–H stretching vibrations from phenolic hydroxyl (-OH) groups. The strong bands at 1700 and 1607 cm^−1^ are attributed to ester C=O stretching and aromatic C=C vibrations, respectively [21]. FTIR spectra of BL films with and without incorporation of TA verified the cross linking or bond formation between the functional groups of TA and the polymers, CS and FG (Figure 1c). The FTIR spectra of the BL films showed major peaks around 3277, 2929, 1635, 1543, 1408, and 1032 cm^−1^, ascribed to N–H (amide A) and O–H stretching, C–H stretching (Aliphatic CH_2_/CH_3_), C=O stretching (amide I), N–H bending and C–N stretching (amide II), and symmetric stretching of COO^−^ and C–O–C stretching of glycosidic linkage, respectively [7]. The above peaks corresponded to the major characteristic peaks of CS-FG.

Compared to the BL film, the FTIR spectra of TA-incorporated BL films had a broader peak around 3276 cm^−1^. Slight changes in the peaks and broadening of the bands indicated the formation of intermolecular hydrogen bonds between TA and the CS–FG matrix. The numerous phenolic OH groups of TA act as potent proton donors and acceptors, forming extensive H-bonds with the -OH, -NH_2_ groups of CS and the -C=O, -NH_2_ groups of FG. Such hydrogen bonding restricted polymer chain mobility, generating a denser network that explains the observed improvements in tensile strength and thermal stability. These non-covalent interactions, mainly hydrogen bonding and secondary hydrophobic forces, were responsible for the enhanced structural cohesion. A slight increase in the amplitude of peaks around 1500–1200 cm^−1^, related to NH_3_ and CO moieties, was found with increasing TA concentration in the BL films (Figure 1c). This could be plausibly due to the changes in secondary structure of FG after introduction of TA in the polymer matrix. From these observations, the film formation of CS-FG in the presence of TA might be influenced by non-covalent interactions, including hydrogen bond formation and hydrophobic interaction.

**Figure 2:**
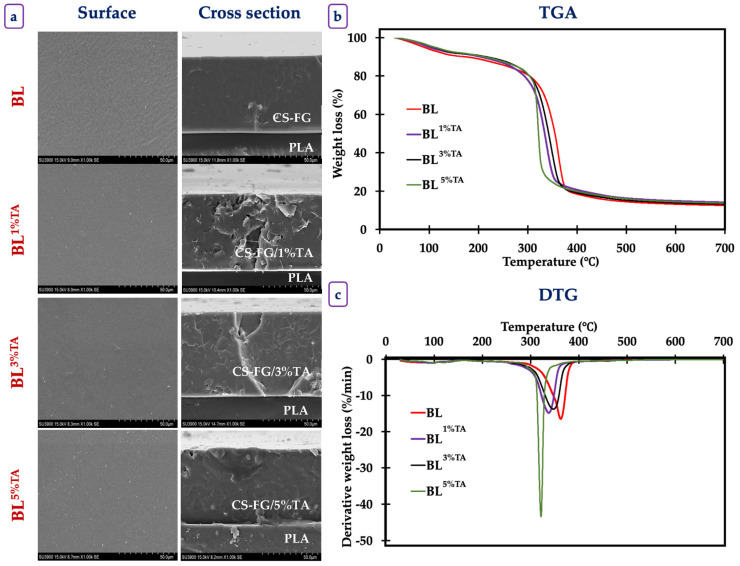
FE-SEM images (**a**) for the surface and cross-section morphology and thermal gravimetric analysis (**b**) and derivative thermograms (**c**) of bilayer films based on chitosan/fish gelatin loaded without and with tannic acid at different levels deposited on polylactic acid film. BL: bilayer films, TA superscript: tannic acid; the numbers in superscript denote the percentage of TA loaded in the CS/FG film.

SEM images of the surface and cross-section of BL films incorporated with TA at varying concentrations are illustrated in Figure 2a. SEM micrographs revealed a smooth, continuous, and homogeneous surface with minor surface irregularities. The micrograph of the BL revealed a relatively smooth, continuous, and homogeneous surface with some minor wrinkles. The cross-section appeared dense and compact without visible phase separation between the polymers and layers, indicating good miscibility of those two polymers and compatibility between TA and the film components. TA particles were uniformly distributed throughout the surface. The surface of the BL films without and with TA had a continuous, intact, and crack-free surface morphology. TA-incorporated films displayed a dense and void-free structure, confirming strong molecular interactions between CS, FG, and TA. Upon the increase in TA (1–5%), the compactness and structural integrity of the BL films were enhanced, as reflected by the smooth surface and uniform cross-section images. The formation of hydrogen bonds between –OH groups of TA, glycerol, and the BL matrix was suggested [22]. Therefore, the inclusion of TA could increase the compactness of BL films without any disruption in the film matrix. Overall, FTIR and SEM analyses confirmed strong intermolecular interactions between TA and the biopolymer matrix, leading to a compact, uniform, and defect-free bilayer structure, which supports the enhanced mechanical and barrier performance of the films.

#### 3.1.4. Thermogravimetric Property of the Films

The thermal analysis of the BL films with varying concentrations of TA provided critical insights into their thermal stability and decomposition behavior (Figure 2b,c). The multi-stage weight loss profile is characteristic of complex multi-component polymeric systems, where each component has a distinct thermal degradation pathway [23].

Three distinct weight loss (Δ*_w_*) stages occurred in different onset temperature (*T_d_*) ranges (Table 3). The first Δ*_w_* (5–7%), observed between 30 and 115 °C, was primarily associated with the evaporation of physically absorbed and bound water within the hydrophilic biopolymer matrix and volatile components [8]. The H-bonded network of these polymers holding water molecules required the energy to be broken down. The second stage of Δ*_w_* (2–3.5%), observed between 110 and 165 °C, could be attributed to the evaporation of residual solvents, the loss of water molecules more strongly bound within the polymer chains, or the very early stages of decomposition of the most thermally labile components, such as the plasticizers (e.g., glycerol) often used in such films. The third stage of Δ*_w_* (~65–70%), observed between 165 and 410 °C, represented the major degradation process, reflecting the thermal decomposition and pyrolysis of the primary polymer backbones. This stage involved the complex breakdown of glycosidic linkages in CS, peptide bonds in FG, ester linkages in PLA, and the phenolic rings in TA. Volatilization of smaller molecules (CO, CO_2_, NH_3_, hydrocarbons) and the formation of char occurred at this stage.

The incorporation of TA into the CS-FG layer induced notable changes in thermal behavior. A clear trend was observed where the weight loss in the first stage (Δ*_w_*_1_) decreased with increasing TA concentration (from 6.98% in BL to 4.89% in BL^5%TA^). This suggested that TA acted as a crosslinking agent within the CS-FG matrix, which was consistent with the mechanical properties and FTIR spectra. The numerous phenolic OH groups in TA were able to form strong H-bonds with the -NH_2_ and -OH groups of CS and FG. This crosslinked network likely reduced the number of available hydrophilic sites that could bind water, thereby decreasing the overall moisture content and water retention capacity of the film. This result was in compliance with the WVP values of the films, which possessed slightly increased water resistance in the presence of TA. The changes in the second stage (Δ*_w_*_2_) were less consistent. BL^1%TA^ showed a slight decrease in mass loss, potentially due to crosslinking, while BL^3%TA^ and BL^5%TA^ had a slight increase. This inconsistency suggested that this stage might be influenced by multiple factors, such as the presence of residual moisture from TA. The third stage (Δ*_w_*_3_) was associated with the polymer decomposition and incorporation of TA, which slightly reduced the overall thermal stability of the polymer blend. TA, a strong crosslinker with a complex structure, began to degrade at temperatures around 200–250 °C [15]. Its incorporation probably reduced the thermal stability of the films, via potentially catalyzing or initiating the decomposition process at a slightly lower temperature. Compactness between polymers might be lowered in the presence of TA inserted between the chains. The crosslinking might also alter the pathway of thermal degradation, leading to an earlier onset [23]. In case of BL^5%TA^, the Δ*_w_*_3_ occurred in a narrower and lower temperature range (165–345 °C) than in the BL (163–410 °C). This could indicate a more synchronized but accelerated degradation process, possibly because the high concentration of TA created a percolated network that could be degraded in a specific pathway, influencing the decomposition of the surrounding biopolymers.

Although TA improved the mechanical properties of CS films, it caused a slight decrease in the maximum decomposition temperature (T_max_), suggesting that the polyphenol could catalyze decomposition reactions at high temperatures [14]. Al-Musawi et al. [24] observed an increase in thermal stability when TA was incorporated in soy protein films. This discrepancy highlighted that the effect of TA was highly dependent on the type of polymer in which TA was incorporated. This was governed by specific interactions via either H-bond or covalent bonds and the polymer’s inherent stability.

#### 3.1.5. Antioxidant Activities of the Films

The antioxidant properties presented in Table 4 demonstrated a profound and concentration-dependent enhancement in the antioxidative properties of BL films upon the incorporation of TA into the CS-FG layer. The ABTS and DPPH assays measure a compound’s ability to donate hydrogen atoms or electrons to stabilize free radicals [25]. BL without TA showed negligible activity, while the incorporation of TA led to exponential increases in activity (*p* < 0.05). BL^5%TA^ exhibited an exceptionally high ABTS-RSA (739.96 mmol TE/g), which was greater than the value for BL^1%TA^ (74.97 mmol TE/g). This indicated that TA, a high-molecular-weight polyphenol, served as an extremely potent electron donor. TA contains numerous galloyl groups, which can provide multiple sites for radical neutralization via hydrogen atom transfer [24]. For DPPH-RSA, the same trend was observed. Nonetheless, the absolute values were significantly lower than those of ABRS-RSA. The disparity between ABTS- and DPPH-RSA could be attributed to the differences in the radical species and reaction kinetics. The ABTS^+^ radical is soluble in both aqueous and organic media and reacts rapidly with most antioxidants. The DPPH^•^ radical is more sterically hindered and larger in size. It was insoluble in the aqueous media [25].

The FRAP assay measures the reducing power of an antioxidant, the ability to donate an electron to reduce Fe^3+^ to Fe^2+^ [25]. The reducing power increased dramatically from 19.74 mmol TE/g in the BL to 78.78 mmol TE/g in BL^5%TA^. This confirmed that TA acted not only as a radical scavenger but also as a potent reducing agent. The electron-donating capacity of its phenolic OH groups is responsible for breaking radical chain propagation reactions commonly observed in lipid oxidation [26]. The MCA assay evaluates the ability of the films to chelate pro-oxidant metal ions, particularly Fe^2+^, which catalyze the Fenton reaction to generate highly reactive OH radicals. The BL film had very low activity (0.29 mmol EDTA/g), indicating minimal innate chelating power of the CS or FG. The addition of TA drastically improved this property, with BL^5%TA^ reaching 5.32 mmol EDTA/g. The ortho-dihydroxy catechol groups in the galloyl moieties of TA are excellent chelators of metal ions [24]. They form stable complexes with Fe^2+^, effectively sequestering it and preventing it from participating in oxidative reactions.

For all assays, the activities were in ascending order: BL < BL^1%TA^ < BL^3%TA^ < BL^5%TA^. This confirmed that the antioxidative capacity was directly imparted by TA and was dose-dependent. Khan et al. [27] documented that CS films incorporated with green tea extract, rich in polyphenols like catechins, had a direct correlation between polyphenol concentration and DPPH-RSA. The films with higher extract loading showed significantly increased activity. Furthermore, FG-based films with TA had dramatic increases in both ABTS- and DPPH-RSA. The film containing the highest TA concentration showed the highest activities. Yu, Wang, Wang, Ge, and Wang [15] developed active packaging using tannins and reported exceptional Fe^2+^ chelation efficiency due to the high density of catechol/pyrogallol groups. Genskowsky et al. [28] reported that maqui berry extract in CS films yielded higher ABTS-RSA than DPPH-RSA. This was attributed to the kinetic limitations and steric inaccessibility of the DPPH radical within the film matrix. Therefore, plant-derived tannins could be used as antioxidants in active food packaging, particularly for lipid-rich products requiring high oxidative stability.

#### 3.1.6. Release Profile of TA from the CS-FG Layer of the Bilayer Film

Controlled release of incorporated bioactive compounds from the polymer matrix is governed by various factors. The physiochemical properties of the active compounds, including the structural properties of the polymer matrix and the surrounding medium where the films are exposed, significantly influence their release patterns [8]. Normally, the release follows either a diffusion process (higher concentration to lower concentration) or desorption (liberation) process of active compounds [29].

**Figure 3:**
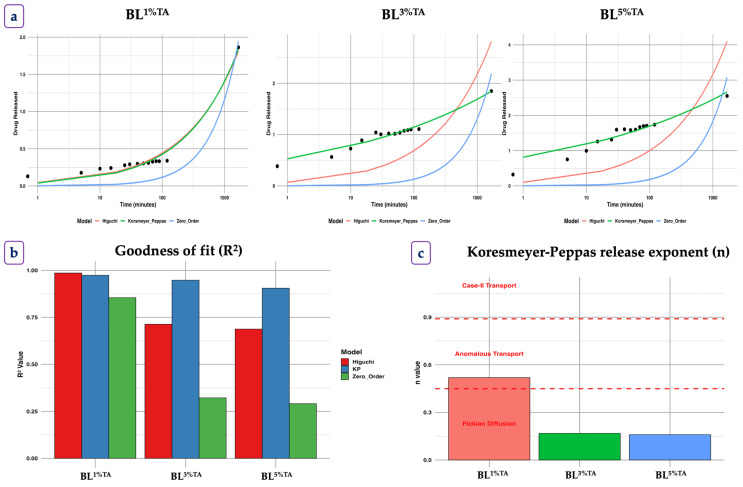
Release profile (**a**), Goodness of fit (R^2^) (**b**), and Koresmeyer-Peppas release exponent (n) (**c**) of tannic acid from bilayer films based on chitosan/fish gelatin loaded with tannic acid at different levels (1, 3, and 5%) deposited on polylactic acid film using release kinetic models including zero-order, first-order, Higuchi, and Koresmeyer–Peppas (KP) models. BL: bilayer films, TA superscript: tannic acid; the numbers in superscript denote the percentage of TA loaded in the CS/FG film.

The release kinetics of TA from the CS-FG film matrix were analyzed using three different mathematical models to elucidate the underlying release mechanisms (Figure 3). To mimic a real food system, TA release was observed in PB at RT. The results indicated that the release behavior did not follow simple zero-order kinetics, as evidenced by the extremely low k values (near zero) and poor fit (R^2^ < 0.5) for all concentrations. The result suggested that the release was not primarily governed by constant dissolution (zero-order). Initially, a rapid TA release occurred within the first 3 h (Figure 3), irrespective of the concentrations. After 3 h, TA release showed controlled diffusion from the polymer matrix following the Fickian model. TA was released rapidly at the beginning, until it achieved an equilibrium state, as regulated by diffusion process. The concentration of release was primarily regulated by the diffusion process compared to the swelling and polymeric matrix relaxation. In contrast, a slower release was observed thereafter up to 50 h. This was explained by the extended hydration of CS and FG, which made the medium more viscous, becoming a gel, thus delaying the quick release of TA. Wang et al. [30] reported a similar pattern of release of EGCG in CS/EGCG films containing bacterial cellulose. Among all film samples, BL^3.00CD^ film had gradual TA release for a longer period of time than other films.

The Higuchi model, which describes Fickian diffusion from a matrix system, showed a slightly improved fit, indicating that diffusion played a partial role in the release process [29]. However, the low R^2^ values across all concentrations suggested that additional mechanisms beyond simple diffusion were involved (Figure 3b). The Korsmeyer–Peppas model, used to analyze complex release mechanisms, provided further insights (Figure 3c). The diffusion exponent (n) values suggested the release was based on Fickian diffusion kinetics (n ≤ 0.45), where the release was primarily controlled by molecular diffusion through the film matrix [8]. Overall, all BL films followed Fickian diffusion with a higher n value, indicating a slightly more complex release profile. This was possibly due to the increased compactness of the film with lower swelling or polymer relaxation at higher TA concentrations [31].

### 3.2. Quality Changes in Baby Clam Edible Portions Packaged in Pouches Made of Bilayer Films Loaded with TA at Various Concentrations During Refrigerated Storage

**Figure 4:**
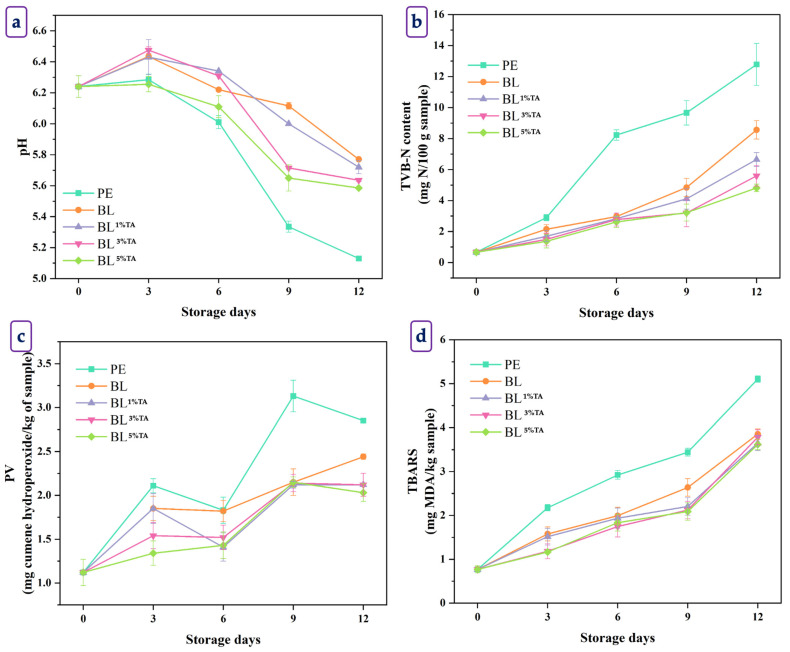
pH (**a**), total volatile base nitrogen content (**b**), peroxide value (**c**), and thiobarbituric acid reactive substances (**d**) of baby clam edible portions packaged in pouches made of bilayer films based on chitosan/fish gelatin loaded without and with tannic acid at different levels deposited on polylactic acid film during the storage at 4 ± 2 °C for 15 days; PE: polyethylene, BL: bilayer films, TA superscript: tannic acid; the numbers in superscript denote the percentage of TA loaded in the CS/FG film. Bars represent the standard deviation (*n* = 3).

The effectiveness of pouches made of BL films incorporated with TA at various concentrations in maintaining the quality of BC-EP was monitored in comparison to that of PE pouches during 12 days of refrigerated storage at 4 ± 1 °C (Figure 4).

#### 3.2.1. Chemical Changes

The initial pH of the BC-EP was 6.24 across all samples (Figure 4a). Over the 12-day storage period, the pH of BC-EP in the control (packaged in PE pouch) exhibited a sharp decline, dropping to 5.13 by day 12, indicating significant acid production (*p* < 0.05). In contrast, all BC-EP packaged in the BL films maintained a higher pH throughout the study. The most effective film, BL^5%TA^, resulted in a final pH of 5.59, which was significantly higher than the control. A decline in pH is typically associated with the accumulation of acidic metabolites, such as lactic acid from post-mortem glycolysis and short-chain fatty acids from bacterial spoilage (e.g., *Lactococcus*, Enterobacteriaceae and *Pseudomonas* spp.) [32]. The significantly slower acidification in BC-EP packaged with the BL pouch, particularly those containing TA, suggested a strong antimicrobial effect. TA is a well-known antimicrobial agent that can disrupt microbial cell membranes and inhibit enzyme activity [33]. By suppressing microbial growth, the TA-containing pouches slowed the production of acidic spoilage metabolites. The higher pH values in the BC-EP of BL group compared to the PE counterpart also showed the inherent antimicrobial properties of CS. This finding aligned with a previous study by Mohebi and Shahbazi [34], who reported a slower pH decline in shrimp packaged with CS-catechin films compared to that packaged in PE, attributed to microbial growth suppression.

TVB-N content, a well-known indicator of protein decomposition and microbial spoilage, started at 0.67 mg/100 g for all samples (Figure 4b). The BC-EP packaged in the PE pouch showed a rapid and linear increase, reaching a very high value of 12.78 mg/100 g by day 12. This value almost reached the proposed rejection limit of 15–20 mg N/100 g for bivalves [35]. All BL pouches effectively slowed the increase in TVB-N content, dependent on the TA concentrations in the pouch. Pouches prepared using BL^5%TA^ were the most effective compared to the others. The lower TVB-N accumulation in the TA-containing pouch provided direct evidence of their potent antibacterial activity. TA effectively inhibited the growth of spoilage bacteria responsible for protein breakdown. Coincidentally, the production of ammonia, trimethylamine, and other volatile bases used as spoilage indicators [7] was also slowed. The concentration-dependent response underscored that a higher loading of TA into the pouch resulted in a greater antimicrobial effect. This result was consistent with the work of Bonilla et al. [36], who found that active packaging with polyphenol-rich ginger essential oil significantly suppressed TVB-N formation in stored fish fillets by inhibiting Gram-negative spoilage bacteria.

The PV, which measures primary lipid oxidation products (hydroperoxides), began at 1.12 meq/kg (Figure 4c). The BC-EP packaged in PE pouches showed a fluctuating but overall increasing trend, reaching a maximum at 3.13 meq/kg on day 9. The bilayer films used for pouch making effectively suppressed the formation of hydroperoxides. The pouch made of BL^5%TA^ film was particularly effective in the early stages and resulted in the lowest final PV (2.03 meq/kg). The significantly lower PV of BC-EP packaged in pouches containing TA, especially on day 3, indicated that TA effectively scavenged initial free radicals, preventing them from attacking fatty acid chains and forming hydroperoxides. The subsequent decline in PV in samples in PE pouches (day 6 to 9) is a typical phenomenon, where hydroperoxides are unstable and decomposed into the secondary products measured by the TBARS assay [37].

The TBARS value, which measures secondary lipid oxidation products (malondialdehyde), started at 0.77 mg MDA/kg (Figure 4d). Lipid oxidation progressed rapidly in BC-EP packaged in PE pouches, soaring to 5.10 mg MDA/kg by day 12. All BL films used for pouch making offered significant protection against oxidation. The BL^5%TA^ pouch was effective in maintaining the lowest TBARS values throughout the storage, and the TBARS value reached 3.62 mg MDA/kg on day 12. The high polyunsaturated fatty acid content of BC-EP makes it highly susceptible to lipid oxidation, leading to rancidity and off-flavors. The superior performance of the pouch made of TA-containing BL films validated their antioxidant properties, as previously stated in Table 4. TA acted as a radical scavenger, donating hydrogen atoms to terminate the free-radical chain reaction of lipid peroxidation [24]. The BL^5%TA^ pouch with the highest antioxidant capacity provided the highest oxidative stability. Berizi et al. [38] demonstrated that CS films embedded with pomegranate peel extract, rich in ellagitannins, effectively reduced TBARS formation in trout fillets during chilled storage. The active films not only delayed the formation of these primary products but also slowed their decomposition into malondialdehyde, as evidenced by the lower concurrent TBARS values. This dual inhibition of primary and secondary oxidation products highlighted the comprehensive antioxidant action of the pouch made of BL film loaded with TA.

#### 3.2.2. Microbiological Changes

**Figure 5:**
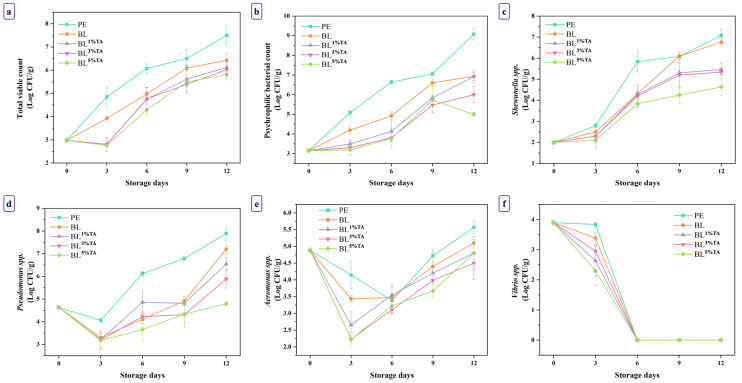
Total viable count (**a**), psychrophilic bacteria (**b**), *Schewanella* spp. (**c**), *Pseudomonas* spp. (**d**), *Aeromonas* spp. (**e**), and *Vibrio* spp. (**f**) of baby clam edible portions packaged in pouches made of bilayer films based on chitosan/fish gelatin loaded without and with tannic acid at different levels deposited on polylactic acid film during storage at 4 ± 2 °C for 15 days; PE: polyethylene, BL: bilayer films, TA superscript: tannic acid; the numbers in superscript denote the percentage of TA loaded in the CS/FG film. Bars represent the standard deviation (*n* = 3).

The microbial quality of seafood is a paramount factor determining its shelf life and safety. Pouches made of PLA/CS-FG BL films incorporated with TA effectively suppressed the growth of multiple spoilage and pathogenic bacteria in refrigerated BC-EP (Figure 5). The antimicrobial efficacy of the pouch upsurged with augmenting TA concentrations. Compared to the PE pouch, all BL pouches with and without TA provided significant microbial inhibition, underscoring their potential as active biodegradable films. Incorporation of TA significantly enhanced the antimicrobial activity of the pouches in a concentration-dependent manner across all microorganisms tested.

The same initial microbial load was found across all samples (~2.98 log CFU/g for TVC and ~3.15 log CFU/g for PBC) (Figure 5a,b). The BC-EP packaged in PE pouches exhibited rapid microbial growth, with TVC and PBC reaching 7.50 and 9.08 log CFU/g, respectively, on day 12. These levels exceeded the generally accepted limit for seafood spoilage (6 log CFU/g) [3]. All BL pouches suppressed the microbial load (*p* < 0.05). The antimicrobial effect was directly proportional to the TA concentration. The BC-EP packaged in BL^5%TA^ pouches successfully maintained the lowest counts throughout the storage period, limiting the PBC to 5.00 log CFU/g and the TVC to 5.82 log CFU/g on day 12. TVC on day 12 was still under the limit. The enhanced effect with TA addition was mainly due to the multifaceted antimicrobial mechanism of this polyphenol. TA can denature vital proteins and inhibit microbial enzymes. It can chelate essential metal ions and disrupt cell membrane integrity [14]. CS film has been reported to have antimicrobial effects, thus extending the shelf life of wrapped foods [7]. The synergistic action between CS and TA created a powerful antimicrobial packaging system in which the microbial deterioration of BC-EP could be slowed effectively. This finding is supported by previous research in which CS films incorporated with TA effectively delayed the growth of total mesophilic and psychrophilic bacteria in raw meat [39].

*Shewanella*, known for producing off-odors, grew rapidly in the BC-EP packaged in PE pouches, reaching 7.08 log CFU/g on day 12 (Figure 5c). The BL^5%TA^ pouches were highly effective in suppressing its growth, maintaining a count at 3.63 log CFU/g on day 12. The results suggest that *Shewanella* spp. were susceptible to the action of the TA released from the pouches. Ding et al. [40] demonstrated that polyphenol application directly inhibited *Shewanella putrefaciens* biofilms and metabolic activity. *Pseudomonas* spp., the prolific psychrotrophic spoilage bacteria, flourished in the BC-EP packaged in PE pouches, reaching 7.89 log CFU/g on day 12 (Figure 5d). The active BL pouches effectively reduced its growth when TA levels upsurged. BC-EP packaged in BL^5%TA^ pouches had a final count of 4.79 log CFU/g on day 12. The significant suppression by the BL^5%TA^ pouches underscored the broad-spectrum efficacy of TA added into the pouch. The mechanism likely involved the inactivation of extracellular proteases and lipases secreted by *Pseudomonas*, which are critical for its spoilage activity. For *Aeromonas* spp. count, BC-EP packaged in BL^5%TA^ pouches also showed a lower final count (Figure 5e). Most notably, for the pathogen *Vibrio* spp., a significant reduction was observed on day 3 in BC-EP packaged in all BL pouches loaded with TA, with the BL^5%TA^ pouch causing a drop from 3.90 to 2.30 log CFU/g (Figure 5f). The initial suppression of *V. parahaemolyticus* was crucial for ensuring food safety. TA has been shown to possess strong anti-virulence properties, potentially reducing the expression of toxins and adhesion factors in pathogenic *Vibrio* spp. [41].

The superior antimicrobial activity of pouches made of TA-loaded BL films was mostly attributed to TA. These TAs possessed abundant phenolic OH and aromatic rings, enabling strong interactions with bacterial membranes, disrupting their integrity and causing leakage of ions and essential metabolites [33]. Structurally, the PLA/CS-FG BL pouches supported sustained release and stabilization of TA. The PLA base layer provided mechanical strength and moisture barrier properties [42], while the CS-FG top layer facilitated intimate contact with the surface of BC-EP and controlled diffusion of TA, extending antimicrobial effects. This multilayer film also reduced oxygen transmission and moisture migration, creating unfavorable conditions for aerobic bacteria [43].

Although a formal sensory analysis was not performed, the chemical and microbiological data could provide the indicators of sensorial preservation. The significant slowing of TVB-N and TBARS formation in clams packaged with TA-containing films indicated a delay in the development of offensive and rancid off-odors [9]. Furthermore, the suppression of key spoilage bacteria like *Shewanella* and *Pseudomonas*, known for producing putrid volatile compounds, suggested odor and flavor stability [40]. The potential sensory impact of astringent and bitter taste from TA was likely mitigated by its controlled Fickian release. Nonetheless, the sensory properties of packed foods remain a key parameter to be investigated for future consumer studies.

#### 3.2.3. Bacterial Diversity of Fresh and Refrigerated BC-EP Packaged in Bilayer Pouches Containing 5% Tannic Acid

**Figure 6:**
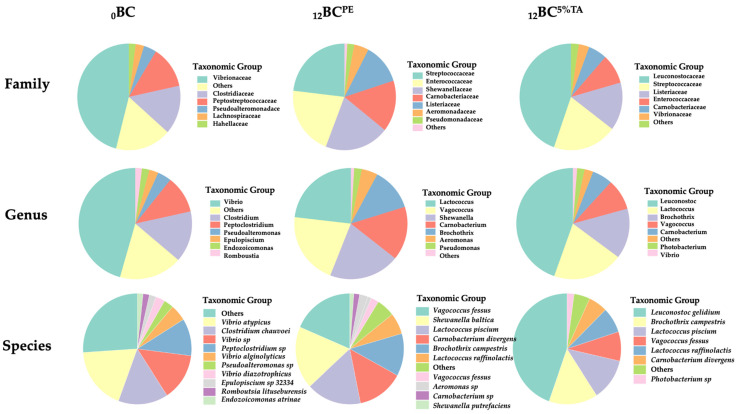
Relative abundance (%) of taxonomic groups at the family, genus, and species levels in baby clam edible portions packaged in pouches made of bilayer films based on chitosan/fish gelatin loaded without and with 5% tannic acid deposited on polylactic acid film before and after refrigerated storage for 12 days. BC: fresh, PE: Polyethylene and TA: tannic acid. Low-abundance species (<1%) and unassigned taxa were grouped under “Others”.

The microbial community, family, genus, and species levels in fresh and packaged BC-EP in PE pouches and pouches made of BL containing 5%TA before and after refrigerated storage for 12 days were analyzed using next-generation sequencing (16S rRNA) (Figure 6). The fresh BC-EP (_0_BC) exhibited a highly diverse microbial profile, which is a typical characteristic of filter-feeding bivalves from an aquatic environment. The high abundance of *Vibrionaceae* was expected, as this family is ubiquitous in marine and estuarine waters and commonly associated with bivalves [3]. _0_BC was dominated by the family *Vibrionaceae* (45.99%) and the genus Vibrio (45.65%), with significant contributions from *Clostridiaceae* (14.85%) and *Peptostreptococcaceae* (12.88%). This high diversity and abundance of taxa like *Photobacterium*, *Endozoicomonas*, and *Epulopiscium* are commonly associated with the filter-feeding bivalve in its natural aquatic environment. This finding was consistent with the NGS result of *Paphia undulata* clams [3]. The presence of *Pseudomonadota*, *Bacteroidota*, and *Planctomycetota* further reflected a complex community derived from the water column and sediment.

After 12 days of refrigerated storage in the PE pouch (_12_BC^PE^), a dramatic change in microbial community was observed. The _12_BC^PE^ community was dominated by Gram-positive, psychrotolerant facultative anaerobes from the families *Streptococcaceae*, *Enterococcaceae*, *Carnobacteriaceae*, and *Listeriaceae*. At the species level, the _12_BC^PE^ community was dominated by spoilage bacteria such as *Shewanella baltica*/*putrefaciens* and *Carnobacterium divergens*, *Vagococcus fessus*, *Lactococcus piscium*, and *Brochothrix campestris*. This group is collaboratively responsible for spoilage during refrigerated storage through proteolysis and the production of volatile amines. *S. baltica* is primarily responsible for the reduction in TVB-N, creating the characteristic fishy off-odor in clams [44]. The emergence of *Shewanella* as a dominant genus is a classic signature of seafood spoilage under refrigeration. The significant presence of Psychrobacter, another psychrotolerant genus, is also a common finding in stored marine fish and shellfish [45]. Previously, Palamae et al. [46] identified *Aeromonas*, *Shewanella*, and *Pseudomonas* as the major seafood spoilage organisms in baby clams.

However, the microbial profile of BC-EP packaged in a pouch made of BL film loaded with 5% TA (_12_BC^5%TA^) was profoundly different, probably due to the antimicrobial action of TA. The suppression of *Shewanella* in _12_BC^5%TA^ resulted in a reduction in volatile sulfides and trimethylamine responsible for putrid off-odors [44]. Similarly, *Aeromonas* was another spoilage bacterial genus, which was completely undetectable. The _12_BC^5%TA^ community was dominated by *Leuconostoc gelidum*/*inhae* (44.63%), a Lactic Acid Bacteria (LAB). Similarly, Sutharsan, Boyer, and Zhao [13] observed a similar dominance of LAB in chilled beef packaged with CS films containing flavonoids. This indicated that TA’s broad-spectrum antimicrobial activity effectively suppressed many bacterial communities, while certain LAB possessed the inherent tolerance mechanisms that allowed them to proliferate. Therefore, the specific suppression of primary putrefactive bacteria directly led to the extended refrigerated shelf life of BC-EP packaged in 5%TA. Previously, Ding, Dwibedi, Huang, Ge, Li, Li, and Sun [40] developed cinnamaldehyde/tea polyphenol/PLA nanofibers that exhibited strong antibacterial activity against *Shewanella putrefaciens*, directly disrupting cell membranes. Similarly, Zhou, Liu, Liao, Wang, and Xia [39] reported that CS/bacterial cellulose films with tea polyphenol nanoliposomes significantly inhibited *Shewanella putrefaciens* and *Pseudomonas* in silver carp.

**Figure 7:**
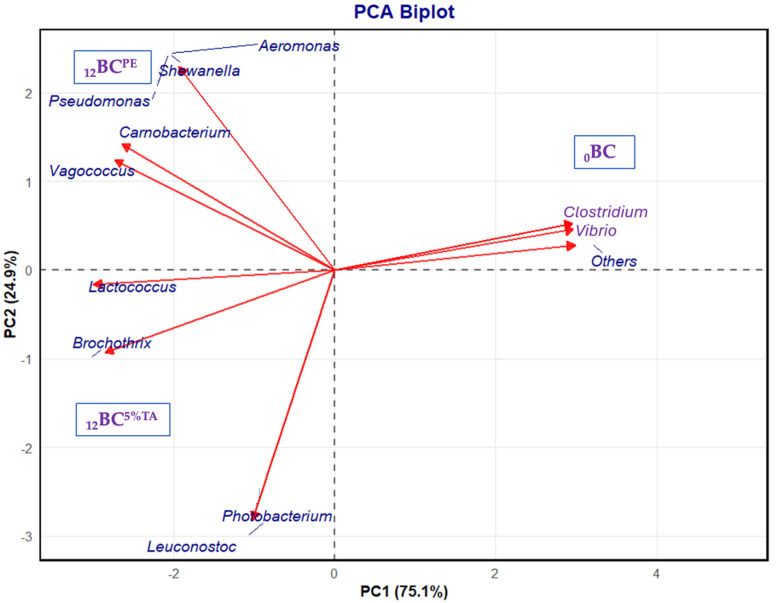
Principal component analysis (PCA diagram) of taxonomic groups at the genus level in baby clam edible portions packaged in pouches made of bilayer films based on chitosan/fish gelatin loaded without and with 5% tannic acid deposited on polylactic acid film before and after refrigerated storage for 12 days. BC: fresh, PE: Polyethylene and TA: tannic acid. Low-abundance species (<1%) and unassigned taxa were grouped under “Others”.

A principal component biplot revealed the beta diversity clusters between the fresh and the BC-EP packaged in PE and BL^5%TA^ pouches after 12 days of refrigerated storage (Figure 7). The two principal coordinates, PC1 and PC2, were 75.10% and 24.90% of the total variance in the bacterial community, respectively. PCA analysis showed that the samples had differences in microbial communities after storage in PE pouches and TA-incorporated BL pouches in comparison to the fresh BC-EP. Refrigerated storage led to increases in spoilage microorganisms such as *Aeromonas*, *Shewanella*, *Carnobacterium*, *Pseudomonas*, and *Vagococcus* [46]. However, the TA effectively inhibited these spoilage bacteria and pathogens and potentially extended the shelf life of the BC-EP.

Alpha diversity indices further confirmed the microbial ecological changes in fresh and refrigerated BC-EP (Table 5). Fresh BC-EP showed immense microbial communities derived from the aquatic environment, many of which are not spoilage organisms. During refrigerated storage, psychrotrophic spoilage bacteria dominate and diminish the other bacteria [45]. BC-EP packaged in PE pouches showed a significant reduction in diversity compared to fresh BC-EP. The Shannon index for _12_BC^PE^ (4.13) was substantially lower than that for _0_BC (5.75), confirming the reduction in original microbial diversity, and the most substantial reduction was observed for _12_BC^5%TA^. This indicated that the TA-containing BL pouch was more effective in suppressing the growth of a wide range of bacteria. Not only was the total number of bacteria reduced, but it also lowered the number of different types of bacteria that could thrive. The Shannon index plummeted from 4.13 (_12_BL^PE^) to 2.88 (_12_BC^5%TA^). This massive ecological shift indicated a much less diverse but more dominant microbial community. The Simpson index, Simpson reciprocal, and Simpson’s evenness values were low for _12_BC^5%TA^. This further confirmed the presence of only a few microbial groups, and other competitors were successfully inhibited. The 5% TA-loaded BL pouch created a potent active packaging atmosphere, releasing TA that severely inhibited the growth of the majority of the spoilage bacteria that normally grow in BC-EP during refrigerated storage. Only the most resistant species survived and proliferated in this hostile environment, leading to a community with very low variety, while some bacteria became dominant.

## 4. Conclusions

The formulated PLA/CS-FG bilayer films incorporated with TA demonstrated excellent potential as active films. The TA loading significantly improved the mechanical properties (from 17.27 to 27.57 MPa), UV-blocking capacity (achieving 100% UV-B blocking at 3% TA), and antioxidant activity of films while maintaining good structural integrity. The films exhibited controlled release behavior, following Fickian diffusion kinetics, enabling sustained antimicrobial activity. When BC-EP was kept at a refrigerated temperature, the samples packaged in pouches made of TA-loaded BL films, particularly at 5% concentration, effectively inhibited microbial growth (reducing TVC by 5-log CFU/g compared to those kept in PE pouches), delayed lipid oxidation, and suppressed spoilage, as indicated by lower increases in both TVB-N content and pH. These protective effects extended the shelf life of BC-EP up to 12 days. NGS confirmed that the 5%TA-containing pouch effectively suppressed specific spoilage organisms, particularly *Shewanella* and *Pseudomonas*, thereby extending the shelf life. This study successfully demonstrated the potential of biodegradable, TA-incorporated BL films as sustainable active packaging for seafood preservation. However, limitations including the increased opacity and dark coloration imparted by TA should be addressed. Future research should focus on optimizing the TA concentration to balance functional benefits with optical and mechanical properties for specific food applications.

## Figures and Tables

**Table 1:** Thickness, tensile strength (TS), elongation at break (EAB), and water vapor permeability (WVP) of bilayer films fabricated from chitosan/gelatin blend loaded with tannic acid at various concentrations deposited over the polylactic acid film.

Film	Thickness (µm)	TS (MPa)	EAB (%)	WVP *
BL	99.96 ± 5.07 ^a^	17.27 ± 1.35 ^c^	85.03 ± 4.86 ^a^	2.52 ± 0.10 ^a^
BL^1%TA^	101.78 ± 2.57 ^a^	17.89 ± 1.41 ^c^	46.73 ± 4.44 ^b^	2.43 ± 0.01 ^a^
BL^3%TA^	103.42 ± 8.08 ^a^	21.63 ± 1.08 ^b^	39.97 ± 2.19 ^c^	2.35 ± 0.29 ^a^
BL^5%TA^	103.50 ± 7.16 ^a^	27.57 ± 1.29 ^a^	38.18 ± 3.07 ^c^	2.30 ± 0.02 ^a^

Values are mean ± standard deviation (*n* = 3). Different superscripts within the same column indicate significant differences (*p* < 0.05). *: ×10^−11^ g m^−1^ s^−1^ Pa^−1^, BL: bilayer, and TA superscript: tannic acid. Numbers represent the various concentrations of tannic acid.

**Table 2:** Color of bilayer films fabricated from chitosan–gelatin containing tannic acid at various concentrations deposited over the polylactic acid film.

Film	L*	a*	b*	*∆E*
BL	89.59 ± 0.06 ^a^	−1.53 ± 0.02 ^d^	3.17 ± 0.09 ^d^	4.25 ± 0.08 ^d^
BL^1%TA^	82.20 ± 1.01 ^b^	2.08 ± 0.16 ^c^	5.04 ± 0.26 ^c^	11.82 ± 0.77 ^c^
BL^3%TA^	80.23 ± 0.59 ^c^	3.65 ± 0.22 ^b^	8.33 ± 0.50 ^b^	15.64 ± 0.80 ^b^
BL^5%TA^	78.63 ± 0.14 ^d^	4.49 ± 0.15 ^a^	10.80 ± 0.24 ^a^	18.48 ± 0.15 ^a^

Values are mean ± standard deviation (*n* = 3). Different superscripts within the same column indicate significant differences (*p* < 0.05). BL: Bilayer film, TA superscript: tannic acid. Numbers represent the various concentrations of tannic acid.

**Table 3:** Degradation temperature (onset temperature, *T_d_* (°C)) and % weight loss (Δ*w*) of bilayer films fabricated from chitosan/gelatin blend loaded with tannic acid at various concentrations deposited over the polylactic acid film.

Film	First Weight Loss		Second Weight Loss		Third Weight Loss	
	Δ*w* (%)	*T_d_* (°C)	Δ*w* (%)	*T_d_* (°C)	Δ*w* (%)	*T_d_* (°C)
BL	6.98	30–112	2.73	112–163	68.45	163–410
BL^1%TA^	6.13	30–114	2.01	114–158	66.71	158–391
BL^3%TA^	4.93	10–108	3.35	108–165	69.89	165–409
BL^5%TA^	4.89	30–109	3.12	109–165	65.05	165–345

BL: bilayer, and TA superscript: tannic acid. Numbers represent the various concentrations of tannic acid.

**Table 4:** Antioxidative activities of bilayer films fabricated from chitosan–gelatin containing tannic acid at various concentrations deposited over the polylactic acid film.

Film	ABTS-RSA *	DPPH-RSA *	FRAP *	MCA **
BL	-	-	19.74 ± 0.23 ^d^	0.29 ± 0.04 ^d^
BL^1%TA^	74.97 ± 9.31 ^c^	4.83 ± 0.01 ^c^	23.51 ± 0.00 ^c^	2.58 ± 0.29 ^c^
BL^3%TA^	280.13 ± 2.12 ^b^	7.18 ± 0.11 ^b^	44.56 ± 0.15 ^b^	4.57 ± 0.35 ^b^
BL^5%TA^	739.96 ± 0.85 ^a^	8.43 ± 0.01 ^a^	78.78 ± 0.15 ^a^	5.32 ± 0.01 ^a^

Values are mean ± standard deviation (*n* = 3). Different superscripts within the same column indicate significant differences (*p* < 0.05). *: mmol Trolox equivalent/g sample, **: mmol EDTA equivalent/g sample, BL: Bilayer film, TA superscript: tannic acid. Numbers represent the various concentrations of tannic acid.

**Table 5:** Alpha diversity of operational taxonomic unit of fresh baby clams and those stored in pouches made of bilayer films based on chitosan/fish gelatin loaded without and with 5% tannic acid deposited on polylactic acid film before and after 12 days of refrigerated storage.

Film Type	Observed Species	PD Whole Tree	Chao	Fisher- Alpha	Shannon	Simpson	Simpson Reciprocal	Simpson’s Evenness
_0_BC	463.40 ± 2.63	26.02 ± 0.16	471.04 ± 4.15	84.75 ± 0.59	5.75 ± 0.02	0.94 ± 0.00	17.95 ± 0.21	0.04 ± 0.00
_12_BC^PE^	98.20 ± 0.92	6.73 ± 0.14	99.08 ± 1.60	13.44 ± 0.15	4.13 ± 0.01	0.91 ± 0.00	10.74 ± 0.06	0.11 ± 0.00
_12_BC^5%TA^	73.70 ± 0.48	7.02 ± 0.01	73.99 ± 0.82	9.65 ± 0.07	2.88 ± 0.01	0.75 ± 0.00	4.05 ± 0.04	0.05 ± 0.00

BC: baby clam; TA superscript: tannic acid, where the numbers denote the concentration (%, *w*/*w*) of TA incorporated into the bilayer film; 0 and 12 in subscripts: fresh clams and clams stored for 12 days packed in bilayer pouches under refrigerated conditions, respectively. Results are presented as mean ± standard deviation based on 10 computational iterations performed for each diversity index.

## Data Availability

The original contributions presented in this study are included in the article; further inquiries can be directed to the corresponding author.
